# Alzheimer’s Disease: Understanding Its Novel Drug Delivery Systems and Treatments

**DOI:** 10.7759/cureus.31394

**Published:** 2022-11-11

**Authors:** Hitaansh Dhingra, Sonali G Choudhari

**Affiliations:** 1 Pharmacology, Jawaharlal Nehru Medical College, Datta Meghe Institute of Medical Sciences, Wardha, IND; 2 Community Medicine, Jawaharlal Nehru Medical College, Datta Meghe Institute of Medical Sciences, Wardha, IND

**Keywords:** anti-inflammatory drugs, rivastigmine, nanomedicines, alzheimer care, alzheimer's disease, alzheimer’s dementia, cognitive enhancements, monoamine oxidase, stem-cell transplant, amyloid plaques

## Abstract

Around the world, there are no fewer than 24 million people who suffer from dementia. Through 2040, this number is expected to rise steadily every 20 years. Alzheimer's disease (AD), the most prevalent kind of dementia, is characterised by a steady deterioration in cognitive function that most often begins with memory loss. Alzheimer's disease (AD) is considered to be one of the leading causes of morbidity in the elderly. Around 5 million people in the United States have Alzheimer's disease. Still, because AD is common in the older and greying population, its prevalence is expected to rise dramatically in the coming decades. As the disease progresses, people with Alzheimer’s disease frequently become dependent on caregivers. The Alzheimer's-diseased brain is characterised neuropathologically by diffuse and neuritic extracellular amyloid plaques, which are often ringed by dystrophic neurites and intracellular neurofibrillary tangles. This particular disease is characterised by the presence of reactive microgliosis and the destruction of neurons, white matter, and synapses. The present review is done to study and learn about new treatments and novel drug delivery systems that may provide benefits to patients with AD. With the new drugs, treatments, constant care requirements, and lost productivity, Alzheimer’s has a substantial financial impact on society. Therefore, better management and therapy are crucial. In this overview, we will briefly go through the current knowledge base about AD, covering the functions of beta-amyloid, tau proteins, and stem cell therapy, and elaborating on novel diagnostic and therapeutic interventions.

## Introduction and background

Alzheimer's disease (AD) is one of the most important medical and social problems impacting older people, both in industrialised and developing nations. For this condition, there are symptomatic drugs that can correct the neurotransmitter imbalance [1]. Various drugs, like cholinesterase inhibitors, function to decrease the breakdown of acetylcholine and are used in the treatment of Alzheimer's and dementia symptoms. In 2022, an estimated 6.5 million Americans aged 65 and older will be diagnosed with Alzheimer's disease. In 2019, there were 121,499 official death certificates recorded, making AD the sixth leading cause of death in the United States. AD is referred to as "early-onset" (or "younger-onset") when it affects someone under the age of 65. The early-onset variant of Alzheimer's disease affects a relatively small percentage of people. When the illness strikes, many of them are in their forties and fifties. After their symptoms manifest, people survive for an average of eight years. However, the disease can advance quickly in certain patients and slowly in others. Treatment of AD consists of cognition-enhancing medications, but no cure exists. Medications and management strategies may temporarily improve symptoms.

## Review

Pathogenesis

Alzheimer's disease is largely caused by abnormal metabolism and the polymerization of usually soluble proteins. When soluble neuronal proteins misfold owing to genetic mutation, environmental conditions, or age, they adopt aberrant conformations and congregate, resulting in dysfunctional neuronal functioning and loss. Extracellular aggregates of A plaques and intracellular aggregations of neurofibrillary tangles consisting of hyperphosphorylated microtubule-associated tau have been reported as histological features of AD. Plaques begin in the basal, temporal, and orbitofrontal neocortex areas of the brain and advance across the neocortex, hippocampus, amygdala, diencephalon, and basal ganglia in later stages [2]. The behavioural and psychological symptoms of dementia often include disturbances in perception, mood, conduct, and mental content. These signs and symptoms, which include apathy, despair, anxiety, psychosis, agitation, and violence, frequently prompt people to seek institutional care. They can also be used as early clinical diagnostic indicators for Alzheimer's disease. In individuals with Alzheimer's, agitation and psychosis are linked to a faster pace of disease development and higher levels of tau phosphorylation [3].

Liposomal delivery systems 

Liposomes are a biocompatible and highly flexible drug delivery system with the ability to transport a wide range of therapeutic compounds over the blood-brain barrier and into the neuronal overlay. They can be customised to increase blood circulation duration and focus on a single or several disease targets [4]. Amyloid beta (Aβ) spontaneously and gradually assembles into amyloid fibrils and oligomers, with the latter being a leading cause of synaptic degeneration and memory problems in AD. However, it is believed that Alzheimer's disease is a complex disease with various processes, including damage to neuronal cells [4]. The blood-brain barrier prevents 98% of potential neuropharmaceuticals from reaching the brain, making it necessary for any effective treatment for Alzheimer's to be transported across it. A biocompatible and highly adaptable drug delivery method, such as liposomes, can pass the blood-brain barrier with a wide variety of bioactive compounds [4]. Different kinds of nanoparticles, such as solid lipid nanoparticles and liposomes, are being explored as potential delivery systems for treating Alzheimer's disease. Targeting the blood-brain barrier's highly expressed receptors or transporters and enhancing stability, such as by using non-natural proteins for peptide medicines to make them resistant to proteolysis, are two prominent strategies for achieving the best possible brain selectivity. As a second option, the active substances might be administered directly into the nose, bypassing the blood-brain barrier. Recent research has started concentrating on liposomes as a potential medication delivery dosage form for treating Alzheimer's disease [4].

Rivastigmine and sympathetic targeted treatment 

Three cholinesterase inhibitors (AChEIs), rivastigmine, donepezil, or galantamine, are considered the first line of drugs for the treatment of mild to severe AD. Another option for treating moderate-to-severe AD is memantine, a noncompetitive antagonist of the N-methyl-D-aspartate receptor [1]. Rivastigmine, an acetylcholinesterase inhibitor, works by increasing the amount of acetylcholine in the brain, which allows nerve cells to communicate. This may help with dementia symptoms. Rivastigmine can be taken orally as pills or liquid, or as a patch applied to the skin [5]. Loaded with rivastigmine liposomes, rivastigmine inhibits brain acetylcholinesterase in a reversible, noncompetitive manner. Oral rivastigmine administration has several drawbacks, including poor stability and a poor capacity to cross the blood-brain barrier (BBB). The administration of rivastigmine via an alternate intranasal route has shown some promise. In an effort to overcome the drawbacks associated with the administration of rivastigmine alone, the addition of a PEGylated polyarginine cell-penetrating peptides (CPPs) derivative to these liposomes increased their stability and aided blood-brain barrier delivery as they have the ability to traverse biological membranes, including the blood-brain barrier [4]. Noradrenaline (NA) as a sympathetic targeted treatment: because the loss of noradrenergic neurons is linked to the onset of Alzheimer's disease, changes in NA may contribute to the disease. Several animal models that reduce NA production using various methods further established the causal link between reduced NA and memory problems, neuroinflammation, and neurodegenerative symptoms of Alzheimer's disease [6]. Monoamine oxidase A and monoamine oxidase B are two monoamine oxidase isoforms that catalyse the oxidative deamination of pharmacologically relevant monoamine neurotransmitters in the outer mitochondrial membranes of most tissues, including the brain. By catalysing the oxidative deamination of biogenic and xenobiotic amines, monoamine oxidase (MAO) plays a crucial role in the homeostasis of neuroactive and vasoactive amines in the central nervous system (CNS) and peripheral tissues [7]. Inhibition of monoamine oxidase will result in a decreased breakdown. Monoamine oxidase plays a significant role in forming amyloid plaques in Alzheimer's patients, and monoamine oxidase B is abundantly expressed in these individuals' brains. Furthermore, consuming too many enzyme inhibitors might result in excessive monoamine oxidase inhibition, which is harmful. Due to serotonin brought on by excessive monoamine oxidase inhibition, serotonin poisoning can result in a dangerous pathological disease characterised by hyperactivity of the serotonin neurotransmitter [7].

Anti-amyloid-β monoclonal antibodies 

The amyloid (A) peptide is the target of the bulk of proposed disease-curing therapies for Alzheimer's disease. Immunotherapy, an incredibly passive vaccination by delivery of exogenous monoclonal antibodies (mAbs), is the most established anti-A treatment strategy. Even though mAb testing has been rife with failure and ambiguous findings, the knowledge collected from these studies has given valuable insights for improved therapies [8]. The accumulation of the amyloid (A) polypeptide in Alzheimer's disease (AD) is thought to be the root of neurodegeneration, synaptic dysfunction, and, eventually, symptoms. Aβ, especially Aβ aggregating blockers and inhibitors of the artificial enzymes gamma- and beta-secretase, is the focus of the vast majority of potential disease-modifying medicines developed in recent years. However, therapy, which combines passive immunisation with the administration of foreign antibodies and active vaccination to stimulate the immune process to deliver its antibodies, is the most advanced anti-Aβ approach [8].

Another aspect which addresses the anti-amyloid hypothesis is β-peptide immunization. The validation of a therapeutic vaccination strategy depends heavily on these transgenic mice. This method includes immunising an animal model of Alzheimer's disease, called PDAPP transgenic mice, with an immunogen called amyloid peptide together with the proper adjuvant, which prevents and reduces the progression of plaque deposition in the brain tissue. In particular, the mice who received the vaccination at a young age showed little to no amyloid plaque accumulation as they aged. Additionally, neuritic dystrophy, a marker of neuronal malfunction, and astrogliosis development were significantly slowed in the treated mice (an inflammatory marker), suggesting that immunisation has benefits for Alzheimer's disease that go beyond just lowering amyloid deposition [9]. Alzheimer's disease clinical trials are now underway for seven monoclonal antibodies, one active vaccination, one antisense oligonucleotide (ASO), and four small compounds targeting components of tau protein biology and neurofibrillary tangle production. The antibodies are primarily focused on extracellular tau, which is transmitted from cell to cell in a prion-like fashion. Several tau monoclonal antibodies, including semorinemab, zagotenemab, and gosuranemab, have failed to show a drug-placebo difference in recent Alzheimer's disease studies [10].

The CSF and interstitial fluid of Alzheimer's disease patients contain amyloid and fibrillary tangles of tau. In humans, amyloid is produced and degraded at a startlingly quick rate. Not only neurons but every cell can create amyloid. When insufficient CSF bulk flow results in extracellular amyloid accumulation, lymphatic mass clearing may provide the necessary and sufficient removal of extracellular amyloid [7]. This demonstrates that a substantial risk factor for developing neurodegenerative conditions may be the glymphatic system's poor activity. Brain microinfarcts activated astrocytes, resulting in the loss of aquaporin 4 (AQP4), which may have decreased and retarded glymphatic CSF influx and accelerated amyloid formation. There is a loop of protein accumulation, glymphatic circuit distortion, further reduction in peptide removal, and disease [11].

Stem cell therapy 

Stem cell therapy, which has implications in the treatment of conditions including Alzheimer's disease, cancer, and blood-related and neurological ailments, is another recently-emerging topic that has drawn much interest. It has shown encouraging results in Alzheimer's disease preclinical studies and early clinical trials, notably in terms of reducing neuroinflammation, enhancing neurotransmission, speeding up neuronal growth, and protein elimination, among several other effects. Many types of stem cells, including embryonic stem cells, induced pluripotent stem cells, umbilical cord blood/placenta stem cells, and mesenchymal stem cells (MSC), can reverse ACh levels, phosphorylated tau, and microglial neuroinflammation. MSC is derived from human leukaemia, bone marrow, and autologous adipose tissue [12].

The ability of the mammalian brain to replace damaged or diseased cells throughout life through neurogenesis and gliogenesis is restricted. Unfortunately, endogenous neurogenesis and gliogenesis suffer considerable declines with age and cannot replace enough lost brain cells to effectively slow the progression of Alzheimer's disease-related neurodegeneration. Whether AD pathogenesis increases or lowers endogenous neurogenesis is currently debated. The damaged central nervous system (CNS) caused by Alzheimer's disease, on the other hand, cannot be repaired by endogenous repair processes. To counteract neuronal loss and cognitive impairment in AD, exogenous neural stem cells may directly replace lost neurons or boost endogenous healing processes such as hippocampus neurogenesis [13].

The first extremely contentious step toward a molecular, cause-focused treatment was taken in 2021 with the approval of an anti-amyloid antibody as a disease-altering therapy for Alzheimer's disease in the United States of America [14]. The unique characteristics of this long-term treatment with intravenous infusions in a particularly sensitive population and a unique side effect profile will provide major implementation issues and call for a high level of cooperation across the healthcare system. These tendencies will probably be furthered by using a multimodal therapeutic strategy in the treatment of Alzheimer's disease [14].

There have been several studies on amyloid-targeted therapies. These include anti-Aβ therapy combinations that work in tandem with newly discovered early identification biomarkers and other therapeutic medicines to address early surgical Alzheimer's disease abnormalities. Expectations and challenges have increased for numerous anti-A drugs in response to the recent FDA approval of a monoclonal anti-Aβ oligomer antibody called aducanumab [15].

Through the modelling of induced pluripotent stem cell-derived (iPSC) brain cells, we have gained the ability to create all of the primary brain cell types from pluripotent cells in less than a decade after the emergence of human iPSC technology. Many critical interactions between brain cells may be recreated using more complicated 3D co-culture systems. These technologies have already made significant contributions to our understanding of human development and illness, particularly neurodegenerative diseases like Alzheimer's. Our capacity to replicate Alzheimer's disease using human cells will increase when these approaches are developed to better resemble in vivo circumstances [16].

Mesenchymal stem cell-derived exosome

Exosomes, a newly emerging method of intercellular communication, provide a unique viewpoint on previously recognised therapeutic interventions for Alzheimer's disease [17]. Exosomes are multivesicular particles released by almost all cell types. Exosomes produced by glial cells or neurons influence cell connections and consequently physiology via conveying miRNAs, proteins, and fats [18]. It has been demonstrated that microglia exosomes may affect the synaptic activity and move neuroprotective substances across cells. By raising the synthesis of ceramide and sphingosine, microvesicles generated from microglia play a part in upregulating synaptic activity. Dysregulation of ceramide and sphingomyelin has been linked to the aetiology of Alzheimer's disease (AD). Additionally, a novel glycoprotein (Synapsin-1) expressed on the surface of Alzheimer’s disease exosomes promotes neurotic outgrowth under oxidative stress, which would be advantageous for the recovery from nerve injury [17]. Exosomes can pass the blood-brain barrier (BBB) and reach brain cells via blood plasma, activating microglia and astrocytes. Exosomes may transfer A or Tau, cytokines, and miRNAs across cells, as well as change the physiology of the receiving cells [18].

Plasma contact system

The plasma contact system, composed of the kallikrein-kinin system (KKS) and the intrinsic coagulation route, may become an essential biomarker. This process has been connected to both epilepsy and Alzheimer's disease. Following cerebrovascular damage, thrombin has been shown to increase excitatory tone and decrease inhibitory tone in CA3 hippocampal neurons, resulting in epilepsy and cognitive impairment. The blood-brain barrier can be broken by kinin release, which can then cause peripheral activation and protein and cell motility from the blood to the brain, starting a downward spiral that increases brain excitability [15].

Anti-inflammatory drugs

The most likely causes of Alzheimer's disease are improper brain wound healing and neuroinflammation. If so, the suggested treatment of intranasal glucocorticoids could be helpful [19]. A transdermal diclofenac patch put on the back of the neck or thoracic spine may also be beneficial in introducing diclofenac into the brain via the cerebrospinal fluid [19]. Following nasal delivery, both pregnenolone and progesterone enter the brain. Pregnenolone and progesterone reduce tau phosphorylation by increasing kinase activity. Regular intranasal insulin improves cognition in Alzheimer's disease, most likely due to its increased concentration in the brain, and the kinetics of intranasal adrenocorticotropic hormone (ACTH) indicate that it enters the brain. Long-term usage of intranasal steroids (particularly earlier-generation steroids) for Alzheimer's disease therapy may increase intraocular pressure (IOP). In contrast, new intranasal steroids (fluticasone, triamcinolone, or budesonide) have negligible effects on intraocular pressure due to their poor bioavailability [19].

According to various records, anti-inflammatory medication users who have inflammatory conditions (such as arthritis) have a decreased chance of developing Alzheimer's disease. As a result, NSAIDs like cyclooxygenase (COX) inhibitors, including aspirin and indomethacin, have been investigated as potential treatments for Alzheimer's disease. Selective COX-2 inhibitors are being studied and clinically tested as a potentially improved therapy for Alzheimer's patients because COX-1 inhibition is known to cause tissue damage in the gastrointestinal tract due to poor cytoprotection. Leukotriene (LTB4), one of the most potent endogenous inflammatory mediators, may be produced more often when COX-2 inhibitors, which decrease the synthesis of one type of eicosanoids, prostaglandins, are used [20].

Neuritic plaques and neurofibrillary tangles, the clinical hallmarks of the disease, are produced as a result of these processes. Although limited to precise support for cognition and related tasks, the therapeutic benefits of the current medical therapy for Alzheimer's disease using anti-dementia drugs (cholinesterase blockers and memantine) are apparent. Over the past 10 years, significant resources have been invested in researching and developing new pharmacological agents with disease-modifying properties. The argument behind this strategy is that by addressing fundamental pathogenic mechanisms in Alzheimer's disease, it may be feasible to slow or even reverse its natural course. Currently, Alzheimer's disease therapy is not just based on the symptomatic effects of cholinesterase inhibitors but also addresses the theories of neurodegeneration of cholinergic neutrons in the brain [21]. Nevertheless, again, current treatments are symptomatic and have some effect on cognitive performance. Efforts to find a disease-modifying therapy for Alzheimer's Disease (A.D.) have focused on therapies that target amyloid (Aβ), but these methods have yet to show a therapeutic effect. Beyond the hypothesis, there are other innovative ways to treat Alzheimer's disease, with neuroinflammation emerging as a particularly active field of study in risk genetic sequencing [22]. Neurological network dysfunction focuses on the control of gamma oscillations and attempts to restore brain tissue through regenerative medicine and new treatment methods for Alzheimer's disease, with a classification based on their primary objectives [23]. These oscillations play an important role in the workings of the brain and are essential for healthy intra-brain communication and basic functioning [24].

Neural networking framework

According to recent research, hereditary and cellular changes cause neural network breakdown, which accelerates cognitive ageing. A novel theory asserts that people with Alzheimer's disease can encode memories but cannot retrieve them. As a result, in addition to therapies that target sick structures, actions that restore neural network connections may be directly helpful in treating memory loss. These therapeutic drugs and therapies also positively respond to the molecular mechanisms that support cellular health restoration [23].

Low-dose radiation therapy

Since low-dose radiation therapy (LD-RT) is beneficial in treating both chronic inflammatory diseases and systemic amyloid deposits, it is possible that it can regulate the amyloid load and cytokine production in Alzheimer's disease. The mechanism for low-dose anti-amyloid radiation therapy’s effect may involve breaking H-bonds and depolymerizing glucosamine glycans, which are highly radiation-sensitive compounds associated with amyloid fibrils. A decrease in leukocyte-endothelial cell interactions and an increase in the synthesis of anti-inflammatory chemicals may be responsible for the anti-inflammatory effect. According to a preclinical study, fractionated regimens of LD-RT significantly decreased amyloid plaques, almost double that of hypofractionated single-dose therapies, which was associated with the control of pro- and anti-inflammatory cytokines and cognitive improvement [25].

Conventional regimens for the treatment of AD

The FDA has authorised four cholinesterase inhibitors (ChE-Is) and one N-methyl-D-aspartate (NMDA) receptor antagonist for treating Alzheimer's disease. Taurine, a ChE, is no longer accessible; the other three are donepezil, rivastigmine, and galantamine. Meta-analyses consistently outperform placebos on measures of function and overall evaluation. In double-blind studies, drug-placebo differences last at least a year. Memantine and cholinesterase inhibitors have comparable effects, with improvements above baseline in cognitive function and transiently stabilising activities of daily living (ADL). Most studies suggest that symptomatic medicines improve present neuropsychiatric symptoms while decreasing the onset of new neuropsychiatric symptoms [16]. A range of additional paths to enhance cognitive performance in Alzheimer's disease are being investigated [26].

γ-Secretase

Research into γ-secretase is very interesting because there is still much to learn about its unique biology as a transmembrane protein complex enzyme. This is because γ-secretase produces A when it cleaves its immediate substrate, amyloid precursor protein C-terminal fragments (APP-CTF), which cause Alzheimer's disease. Understanding how γ-secretase involves breaking it down into over 100 targets and how various signalling cascades could result in various physiological activities in Alzheimer's disease will take time. It is also critical to comprehend how γ-secretase modulators (GSMs) and -secretase modulatory proteins (GSMPs) influence various substrate processes. This blocks APP processing in particular. This information could aid in the creation of medications that change Alzheimer's disease [27].

Epigenetics 

The decreased histone methylation results in dysfunctional synaptic transmission, neuronal growth, and memory. DNA methylation controls the expression of genes related to Alzheimer's disease under the action of related enzymes. Changes in related enzymes result in a decrease in histone methylation, which results in the inactivation of memory-related genes and abnormal p53 levels [28]. Through its interactions with cellular factors, viral factors, and RNAs that can promote the development of various disorders, p53 plays a vital role in the progression of neurodegenerative diseases. As a result, inhibiting p53 may be an appropriate target for restoring neural functioning [29]. Inhibitors of histone acetyltransferases (HATs) and histone deacetylases (HDACs) can restore these alterations and prevent Alzheimer's disease [28]. Increased phosphorylation of histones is linked to the clinical phenomenon of Alzheimer's disease, resulting in memory impairment.

Some miRNAs can promote synaptic and cognitive dysfunction, while others can prevent neuronal death and degenerative damage and raise the I.Q. of animals with Alzheimer's disease. Ubiquitination alterations can result in A deposition and cause neurodegeneration. Because of the specific activity of miRNAs during Alzheimer's disease, several miRNAs have also been proposed as blood markers for the early detection of Alzheimer's disease. The diagnosis and treatment of Alzheimer's may involve modifying the factors linked to the onset of the illness in persons with the condition. Early in the course of the disease, epigenetic changes may impact the activity of relevant genes [28].

Superparamagnetic iron oxide nanoparticles

There is some promise in using magnetic nanoparticles to treat or prevent Alzheimer's disease. In a groundbreaking discovery, Loynachan showed that magnetic particles might be used to destroy amyloid clumps by harnessing their magnetic characteristics. This is a preliminary demonstration of the feasibility of using nanoparticles and an alternating magnetic field to modify Alzheimer's disease pathogenesis. Even though this treatment approach's use in humans has not yet been thoroughly studied, we may still see synergy for both clinical and therapeutic goals [30].

Enhancing autophagy through drug repositioning

Three antihyperlipidemic medications-gemfibrozil, simvastatin, and (icosapent ethyl) vascepa-are now being researched for use as the medication for Alzheimer's disease. A way that gemfibrozil increases Aβ clearance has been hypothesised to be through the stimulation of autophagy. Statins are frequently used to treat hyperlipidemia, but over time, substantial evidence has shown that their therapeutic effects may come from sources other than their capacity to decrease cholesterol [31]. It has been acknowledged that conventional approaches to drug development (such as single target, single drug) fall short of meeting the unmet demand for efficient treatments for the management of complicated multifactorial disorders like Alzheimer's disease. On the other hand, due to medication-drug interactions, combination treatments that focus on several distinct targets may increase drug toxicity. A careful examination of distinct but overlapping disease-pathological pathways may yield novel approaches to medication repurposing [31]. 

Traditional Chinese medicine

Chinese herbs may inhibit the signalling pathways linked with the degenerative progression of Alzheimer's disease, such as Nrf2, Janus kinase (JAK)/signal transducer and activator of transcription (STAT), the ubiquitin-proteasome pathway, and an autophagy-lysosome path that leads to the peroxisome proliferator-activated receptors (PPARs) pathway. The JAK/STAT signalling system regulates various CNS activities, including neurogenesis and gliogenesis. They might avoid oxidative damage, manage ubiquitin-proteasome system activity, regulate interplay across channels, keep the balance of inflammatory response interactions, adjust autophagy, and finally enhance cognitive deficits in Alzheimer's disease patients [32].

Chalcones as potential ligands 

Chalcones have a wide range of biological action since they are the building blocks of all known flavonoids. Adenosine A2A and A1 receptor affinity, a strong capacity to inhibit -syn aggregation, and a high ability to activate Nrf2 signalling have all been demonstrated. They also have a high inhibitory potential on the monoamine oxidase B, catechol-O-methyltransferase, and acetylcholine enzymes. Simple alterations within the A and/or B rings can produce such consequences [33].

Low-dose ionizing radiation 

Low-dose ionising radiation (LDIR) has recently attracted much attention from academics and physicians as a practical therapeutic approach for treating neurodegenerative illnesses. In fact, the issue of whether LDIR exposure harms one's central nervous system (CNS) remains relevant today [34].

Caffeine

Caffeine was found in studies to draw water and promote plaque expulsion from the membrane, which led to obvious amyloid fibrils as seen by microscopes and x-ray diffraction. The particular mechanism of caffeine's neuroprotective impact on Alzheimer's disease is unknown. However, it is known that membranes have a critical role in the first phases of peptide aggregations, where they act as a solid foundation for the crosslinking of adjacent Aβ molecules. As a result, coffee avoids crosslinking with adjacent monomers by expelling peptides early [35]. Caffeine is neuroprotective against dementia and perhaps Alzheimer's, although further research is needed to confirm this association. According to various trials, caffeine is a cognitive stabilizer, different from an enhancer. Although clinical studies suggest that gender may complicate the neuroprotective impact of coffee, they are not definitive. It is also discovered that substantial evidence from studies indicates caffeine has several beneficial effects in Alzheimer's disease models, but more research is required to pinpoint all the mechanisms underlying its neuroprotective properties. Although caffeine in high doses can cause insomnia, it also increases the risk of neurodegeneration [35]. 

Role of gut microbiota 

In terms of modulating gut microbiota over a lifetime, the diet has the greatest influence. It is reasonable to expect that dietary treatments such as a diet high in fruits and vegetables and low in saturated fats will help reduce cognitive decline while also improving neuroprotective functions. The resulting changes in gut microbiota composition may one day serve as an additional strategy to prevent or manage dementia, given that, as was previously indicated, Alzheimer's disease is related to microbiota. There is relatively little clinical research examining the interactions between gut microbiota, nutrition, and neurodegeneration, making it difficult to establish a cause-and-effect connection between these factors [36].

Nanomedicines

Numerous studies have been conducted to determine the best strategy for stopping the aggregation and fibrillation of Aβ in Alzheimer's disease. The development of insoluble amyloid fibrils from the buildup of A peptides is one of Alzheimer's disease's most conspicuous pathological characteristics. Inhibitors of Aβ aggregation may be helpful for Alzheimer's disease. Various medications can destabilise the A fibrils in vitro, preventing A aggregation and neurotoxicity [37]. The functional nanoparticles (NPs) may successfully suppress protein aggregation. By using peptide-containing, light-activated gold nanoparticles (AuNPs), preformed fibrils may be broken apart. When the nanocarrier's surface is correctly functionalized, damaging ions cannot escape from the nanocarrier. Tiny fibres may be broken up by NPs, preventing them from accumulating [37].

Neuroprotective effects of carvacrol

One of the main components of the essential oils of various plants, such as Thymus, Zataria, and Lippia of the Verbenaceae family, is carvacrol (5-isopropyl-2-methyl phenol), a naturally occurring monoterpenoid phenol. Carvacrol may be a helpful therapeutic agent for neuroprotection and the improvement of cognitive function, according to the research that is currently accessible. Its multifunctional properties, which include antioxidant, anti-inflammatory, and AChEI properties, maybe the reason for this. Additionally, carvacrol contains a lot of neuroprotective properties, such as anti-ischemic and anti-epileptic properties [38].

Glymphatic and the impact of osteopathic manipulative treatment 

A fluid known as lymph flows through the brain's interstitium and, in diseased situations, abnormally gathers and facilitates the accumulation of other toxic substances. The glymphatic system deals with the circulation, buildup, and clearance mechanisms of metabolic waste [36]. Aquaporin-related channels control brain fluid flow, removing metabolites, oedema, and waste. However, this fluid accumulates during illness, whether as a result of structural abnormalities, aquaporin channel downregulation, the accumulation of toxic compounds, or the body's inability to properly discharge the interstitium [39].

## Conclusions

The cerebrospinal fluid (CSF) and chalcone ligand biomarkers of amyloid (A) and tau proteins are highly precise in identifying the pathophysiological and neuropathological changes of AD and are examples of biomarker identification and validation advances in Alzheimer's disease. Nasal steroids, rivastigmine, and aripiprazole are the pharmacological agents used in the treatment of Alzheimer's disease. Stem cell therapy and epigenetics have shown promising results in the field of biotechnology in medicine. The first line of drugs remains cholinesterase inhibitors (AChEIs) such as rivastigmine, donepezil, or galantamine, but the aspect of anti-amyloid is of great importance as well. In the coming years, more research will be focused on the pathogenesis of amyloid and tau. Although there is no absolute cure for this disease, several novel drug delivery systems and strategies are proving to be game-changing. If not completely curable, symptoms and severity can be managed by the above-discussed treatments and drug delivery systems. With the increase in deaths due to AD and the exponential growth in the number of cases, more advancements and a better understanding of these novel drug delivery systems and therapeutic strategies would benefit the world a lot.
